# Systems Level Dissection of *Candida* Recognition by Dectins: A Matter of Fungal Morphology and Site of Infection

**DOI:** 10.3390/pathogens4030639

**Published:** 2015-08-21

**Authors:** Lisa Rizzetto, Tobias Weil, Duccio Cavalieri

**Affiliations:** 1 Research and Innovation Center, Fondazione E. Mach, via E. Mach 1, San Michele all’Adige (TN) 38010, Italy; E-Mails: lisa.rizzetto@fmach.it (L.R.); tobias.weil@fmach.it (T.W.); 2 Department of Neuroscience, Psychology, Drug Research and Child Health (Neurofarba), University of Florence, Firenze 50139, Italy

**Keywords:** *Candida albicans*, innate immunity, morphology, C-type lectin receptors, pathogen, yeast-hyphae

## Abstract

*Candida albicans* is an ubiquitous fungal commensal of human skin and mucosal surfaces, and at the same time a major life-threatening human fungal pathogen in immunocompromised individuals. Host defense mechanisms rely on the capacity of professional phagocytes to recognize *Candida* cell wall antigens. During the past decade, the host immune response to *Candida* was dissected in depth, highlighting the essential role of C-type lectin receptors, especially regarding the power of the Dectins’ family in discriminating between the tolerated yeast-like form of *Candida* and its invading counterpart, the hyphae. This review focuses on the immuno-modulatory properties of the *Candida* morphologies and their specific interactions with the host innate immune system in different body surfaces.

## 1. Introduction

Pathogens are usually regarded as autonomous causative agents in virulence studies even though the host and the host microbiota can influence their pathogenicity [1]. In healthy individuals, most of the microbial resident eukaryotes are rather mutualistic or commensal and do not cause infections. However, when the host microbiota gets disturbed or the host defense mechanisms extenuated these commensals can become pathogens [2]. An important representative of such opportunistic fungi is the yeast *C. albicans,* a permanent member of the human microbiota, present on the skin [3], oral cavity [4], vagina [5,6] and gut mucosa [7] in 30%–70% of healthy individuals. Under normal conditions, *C. albicans* does not cause significant disease [8,9], yet, it has the potential to infect and colonize the host in immunocompromised individuals, like HIV-patients [10], lung transplant recipients [11] and cystic fibrosis patients [12]. Invasive *Candida* infections lead to high mortality and to enormous treatment costs of annually US$ 8 billion alone for the United States [13].

Generally, the interaction between the human host and its microbiota is based on the immunological tolerance of colonizing microorganisms by the mucosal host defenses. This commensal stage is regarded to be inoffensive to the host due to a tight control that is highly regulated by a continuous or transient cross-talk between the fungus and the host immune system [14]. The latter maintains homeostasis with the resident mycobiota, ensuring the balance between tolerogenic and pro-inflammatory response. 

In parallel, disturbance of the network of competitive commensal bacteria can lead to increased susceptibility to *C. albicans* infections and distorted immunological responses against *C. albicans* have been proposed to contribute to the pathological auto-inflammation that occurs in patients with Crohn’s disease [15].

**Figure 1:**
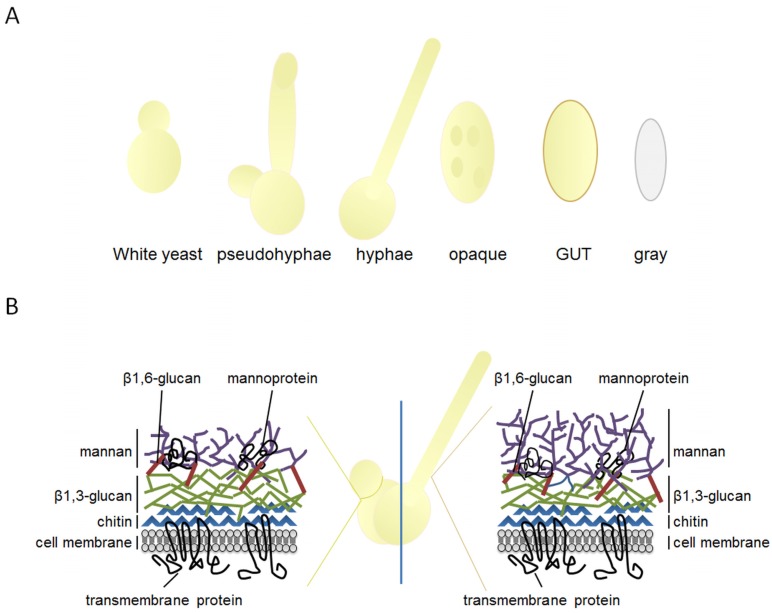
*Candida albicans* morphotypes and cell wall composition. (**A**) The various morphologies of *C. albicans* play different roles in colonization and disease. White yeast cells are important for establishment and dissemination of fungal infections, and pseudohyphae and true hyphae for tissue penetration and invasion. Opaque-phase cells are mating competent cells that resemble gastrointestinally induced transition (GUT) cells, but other than opaque cells they lack surface pimples in their cell wall and have been uniquely adapted for commensal growth; (**B**) Schematic diagram of the cell wall composition in *C. albicans* yeast cells and hyphae. In yeast and hyphal forms the sugar moieties present on the fungal cell wall are the same but their exposure and thickness determine different immunogenic properties. For example, β-glucans are just exposed on the yeasts’ bud scars and division septa and are hidden by a surface layer of mannan in the hyphal form.

*Candida* is a dimorphic fungus with distinct phenotypical features in its yeast form *versus* its hyphal form. This phenotypic transition led to a long-lasting debate on relative attributes of *C. albicans* morphotypes during the colonization of skin and mucosae, and subsequently on the invasion of the bloodstream and deep tissues [16,17]. Whereas disseminated candidiasis is generally induced by the yeast form, mucosal diseases are more strongly correlated to filamentous forms like invasive hyphae and pseudohyphae [18]. The *Candida* cell wall is mainly composed of different carbohydrate moieties, such as β-glucan, mannan and chitin (Figure 1). In the course of a *C. albicans* infection the initial response of the innate immune system is determined by the recognition of these fungal cell wall components, also referred as pathogen associated molecular patterns (PAMPs), by pattern recognition receptors (PRRs) located on the surface of innate immune cells. C-type lectin receptors (CLRs) are the main group of PRRs involved in antifungal responses, recognizing polysaccharide structures of microorganisms [19]. Individual CLRs, particularly receptors of the “Dectin-1” and “Dectin-2” families [20], are able to recognize different fungal pathogens but there is a clear overlap in the substrate recognized by some of these receptors [19,21,22,23].

Differences in TLR and CLR signaling can be used to classify microorganisms for the induction of pro-inflammatory or tolerogenic signals [24,25]. This fine-tuned recognition potential opens the possibility to investigate in depth the human susceptibility to fungal infections caused by specific strains. Thus, discovering differences and commonalities allows the subsequent development of new antifungal therapies.

**Figure 2:**
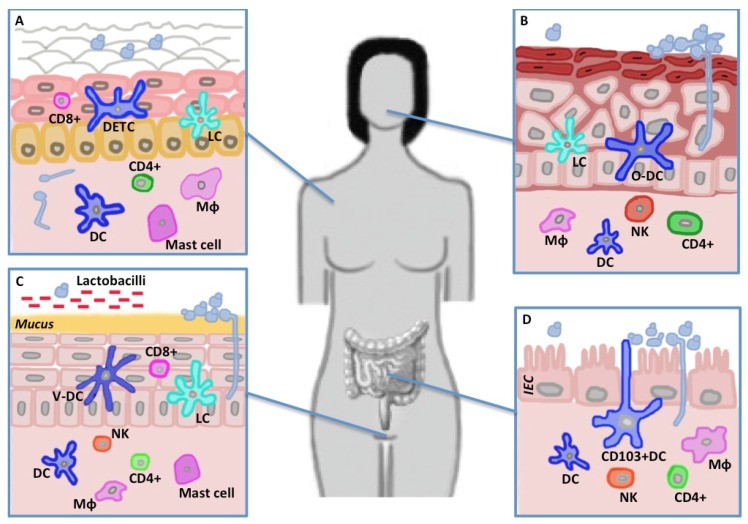
*C. albicans* and mucosal immunity. The major cells participating in the Dectin-1 mediated antifungal immune response are depicted for four body sites frequently subjected to *Candida* infections. (**A**) Skin: On the epidermis *Candida* is mainly present as yeast-like cells while pseudohyphae are the predominant form in the dermis. The epidermis is interspersed with LCs, DETCs and CD8+ T cells, representing the first line of defense towards yeast-like cells. To face hyphal invasion, the dermis contains CD4+ T effector memory cells, macrophages, mast cells and DCs; (**B**) Oral cavity: DCs and LCs reside in the stratified squamous epithelium. In the lamina propria, DCs project dendrites into the epithelium to uptake antigens and to prime CD4+ T cells; (**C**) Vagina: In healthy individuals *Lactobacillus* species produce a low pH which keeps pathogens at bay. The vaginal mucosa is surveyed by macrophages and DCs present in the epithelial layer (LCs, CD8+) and the submucosal lamina propria (CD4+); (**D**) Gut: In the gut lumen *C. albicans* is predominately present in small numbers and as yeast-like cells which do not cause epithelial damage. Contrary high loads of *Candida* lead to abundant growth on the surface and the mucosa is invaded by the hyphal form. CD103+ DCs, macrophages and NK cells collaborate in promoting the activation of the appropriate Th response. Mϕ, macrophage; DC, dendritic cell (prefix “O” oral, “V” vaginal); LC, Langerhans cell; DETC, dendritic epidermal T cells; NK, natural killer cell; CD, cluster of differentiation.

In this review, we will describe the most recent discoveries on the immuno-modulatory properties of *C. albicans* morphotypes relevant to the interaction with the host, and the immunological consequences of the underlying shifting nature of the cell surface of this fungus. We will further focus on new advancements regarding the important role of the Dectin-1 and Dectin-2 receptor families in terms of antifungal mucosal immunity in different body districts (Figure 2).

## 2. The Highly Plastic Nature of *C. albicans* Morphology and Its Implications for Pathogenicity

As indicated, *C. albicans* uptake and clearance require a concert action of different receptors of the host innate immune system which is responsible for the recognition of PAMPs and for the subsequent release of an antifungal-specific immune response. *C. albicans* developed an enormous phenotypic armamentarium to colonize diverse host niches and to escape the host immune defense. A key virulence factor of *C. albicans* pathogenesis is its ability to switch between different morphologies, comprising yeast, pseudohyphae and true hyphae (Figure 1). *C. albicans* uses the reversible transition to filamentous growth as a response to environmental cues and to either block or avoid recognition by stimulatory PRRs [26].

The current paradigm is that yeast forms are critical for colonization, early infection and dissemination, while filaments are responsible for tissue invasion and deep infection [26,27]. These morphological transitions go along with cell wall remodeling, leading to a change in the composition of the exposed components, which in turn influences the recognition by the host immune system. The fungal cell wall consists of two main parts: an inner skeleton composed of β-glucan and chitin and an outer cell surface layer of mannan (Figure 1). Cell wall carbohydrates represent the major PAMPs of *Candida* and their different exposure on the surface is thought to influence the recognition by leukocytes. Here, mannan, as the outermost layer, masks the subjacent cell wall layers and thus, prevents the Dectin-1 pro-inflammatory response [28,29], yet on the bud scars chitin and β-glucan can become exposed to the surface [30,31].

Despite this findings, recent analyses of the interaction between murine macrophages and *C. albicans* mutants defective in filamentation suggest that rather than filamentation, the change in the cell wall composition, that occurs during the yeast-to-hyphae transition, is the driving force for infection causing macrophage lysis and *Candida* outgrowth [32]. Here, a genome scale analysis revealed 102 negative and 872 positive genetic regulators during *C. albicans* morphogenesis highlighting the importance of the early and middle stages of ergosterol biosynthesis during adaption or evasion of the immune system [32].

Further, macrophage migration towards *C. albicans* has been shown to depend on the glycosylation status of the fungal cell wall, but not on cell viability or morphogenic switching from yeast to hyphae. Lacking specific PAMPs, *C. albicans* glycosylation mutants are slowly phagocyted by macrophages and in particular mannosylation seems to be a key determinant in the rate of macrophage engulfment, consequent fungal recognition and clearance through differential activation of macrophage PRRs [33].

Additionally, apart from protection against the host defense by camouflaging cell wall polysaccharides against detection of the innate immune system, covalently linked cell wall proteins (CWPs) serve several important virulence features such as adhesion, biofilm formation and tissue invasion. The diversity of CWPs is amplified by another particularity of *Candida* species: the ambiguous translation of the serine CUG codon as serine and leucine [34,35]. Here, especially CWPs are enriched in CUG codons and it was shown that increased CUG mistranslation is an important factor in triggering the above mentioned virulence attributes by creating variability in CWPs [35,36]. These studies on codon ambiguity open a new perspective on the immune-modulatory capacities of *Candida* by expanding its adaptation to host microniches [35,36].

Another fascinating transition is the mating-type locus (MTL) controlled white-opaque switch from the yeast morphology to the mating-competent elongated cell morphology with pimpled cell walls [37]. This switch is a phenotypic transition associated with changes in cell morphology, physiology and gene expression [38] and is accompanied by differences in virulence. In mouse models the smooth white cells have been shown to colonize mouse kidneys rapidly and at higher levels than the hyphae-like opaque cells, while the latter have advantages in the colonization of the skin [38]. Recently two new cell types, gray and GUT (gastrointestinally induced transition), were added to the *C. albicans* phenotypic switching system [39,40]. GUT cells resemble opaque cells but lack pimples on their cell surface. They seem to be optimized for commensalism in the mammalian digestive tract [40]. Gray cells are also elongated but smaller than opaque and GUT cells and colonies have a smooth gray colored appearance [39]. Both cell types showed higher expression of secreted enzymes and cell-wall remodeling proteins when compared to white and opaque cells. Interestingly, gray cells had an advantage over white cells regarding dissemination in an *ex vivo* tongue infection model and caused more damage in an *in vivo* skin infection model [39].

Further, *C. albicans* forms biofilms which represent a mixture of yeast and filamentous cells, but also in this regard *C. albicans* is unique as it forms two types of biofilms, one pathogenic and one sexual [41]. Like for white-opaque switching the form of the biofilm depends on the MTL conformation. Ninety percent of strains exhibit the a/alpha MTL configuration forming the pathogenic biofilm that is robust, impermeable and drug-resistant, while homozygosity of the MTL leads to a drug-susceptible biofilm that is associated to mating [41]. Biofilm formation starts with the adherence of yeast-form cells to the substrate but the formation of “sticky” hyphae is regarded as the initiation step of biofilms [42]. For dispersal the yeast to hyphae transition seems to be reversed as mature biofilms release yeast-form cells and this dispersed cells exhibit an increased potential for adherence and filamentation. Therefore, *C. albicans* biofilms in implanted medical devices are a main source of disseminated infection leading to high mortality [42].

Taken together this complex plasticity and strong remodeling ability together with the discovery of novel cell types enable a specific capability of *C. albicans* to alternatively mask or uncover immune-stimulatory antigens, and thus, provides an incredible complexity of host—fungal interactions.

## 3. C-Type Lectins in Fungal Immunity: Dectin-1 and Dectin-2 Signaling

**Figure 3:**
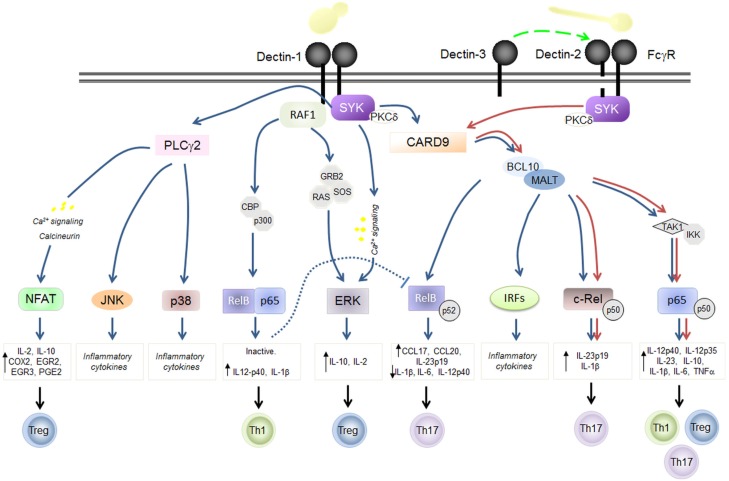
Schematic representation of selected signal networks induced by Dectin-1 and Dectin-2. Dectin-1 and Dectin-2 induce intracellular signaling via tyrosine (Y)-based activation motifs (immunoreceptor tyrosine-based activation motifs or ITAMs) which recruit and activate Syk-kinase either directly, or indirectly through the FcγR adaptor chain. Signaling through protein-kinase C (PKC) δ activates the Card9-Bcl10-Malt1 complex and induces gene transcription and the production of various inflammatory mediators. Dectin-1 can signal via the Raf-1 kinase pathway which modulates other signaling pathways, including those induced by the Toll-like receptors (TLR) and the Dectin-1/Syk pathway. Dectins can also collaborate with TLRs, to synergistically induce or repress various cytokines and chemokines and to mediate fungal phagocytosis (not shown). Dectins-mediated recognition of *C. albicans* yeasts or hyphae drives their uptake and killing by phagocytes, and directs the development of protective Th1/Th17 responses. Induction of IL-12 drives IFN-γ production by Th1 cells, which is critically required for the activation of phagocytes. Signals inducing IL-10 production lead to Treg differentiation. On the other hand, induction of IL-1β, IL-6, and IL-23 promotes Th17 differentiation, which drives the production of IL-17 and IL-22.

CLRs receptors play a major role in anti-fungal immunity, both in mice and men [43]. Receptor-mediated responses include fungal binding and phagocytosis, as well as production of soluble molecules, including cytokines, chemokines and inflammatory lipids, which drive inflammation and adaptive immunity in the host. These actions are dependent on the activation of a common signaling pathway that involves Syk kinase, Card9 and NF-κB in both DC and macrophages [44] and that directly modulate adaptive immunity by priming Th1 and Th17 responses [19,20,21,22] (Figure 3). Indeed, defects in several components of the Th17 pathway, ranging from the signaling molecules (CARD9, STAT1, STAT3) to the cytokines involved (IL-17), have been linked to chronic mucocutaneous candidiasis [23,45]. All of these responses are mainly driven by the Dectin-1 and Dectin-2 receptors [43].

### 3.1. Dectin-1

Dectin-1 signaling is considered archetypal for non-Toll like pattern recognition receptor mediated signal transduction. It was first identified on DCs [46] but later it was shown that the receptor is also widely expressed on other cell types like macrophages, neutrophils (Robinson, *et al.*, 2006, Taylor, *et al.*, 2002), γδ T cells, and on epithelial cells of the mucosa (Martin, *et al.*, 2009, Rand, *et al.*, 2010). Dectin-1 recognizes β-1,3-glucans in a calcium-independent manner, and is involved in both ligand uptake and phagocytosis, as well as proinflammatory cytokine production.

Through its pathway, Dectin-1 can synergistically collaborate with the TLRs to induce pro-inflammatory responses (Brown, 2006) but also without TLR engagement it can elicit a robust inflammatory response in DCs [19]. This response is modulated by the progression of the phagocytosis following Dectin-1 clustering into “phagocytic synapses” (also known as phagocytic cups) [47], resulting in a dramatically augmented inflammatory response which is mainly induced by β-glucan through a mechanism previously referred as “frustrated phagocytosis” [48,49]. Frustrated phagocytosis indicates for example the limit of phagocytes to internalize just as many particles that they burst [50].

Similar to other CLRs, Dectin-1 engagement leads to tyrosine phosphorylation of the ITAM-like/ITAM motifs, recruitment and activation of Syk kinase and subsequent activation of the CARD9–Bcl10–Malt1 complex through PKCδ [51,52] (Figure 3). Stimulation of this pathway by Dectin-1 and other Syk-independent pathways, as those mediated by Raf-1, results in the activation of several transcription factors including canonical and non-canonical subunits of NF-κB as well as NFAT, IRF1 [53,54,55] and IRF5 [56]. A recent study in mice showed that, Dectin-1 activation of CARD9 recruits and activates HRas through Ras-GRF1 phosphorylation resulting in the induction of ERK-mediated signaling in bone-marrow derived macrophages and DCs [57]. This CARD9 activation lead to an increased survival of mice during systemic candidiasis [57].

In general, Dectin-1 signaling regulates numerous cellular responses like phagocytosis, autophagy and the respiratory burst as well as, the production of inflammatory lipids, cytokines and chemokines such as the Th17- polarizing cytokines IL-23, IL-6 and IL-1β. Especially, Dectin-1 induced production of IL-1β is of interest, as it involves both the NLRP3/caspase-1 and non-canonical caspase-8 inflammasomes [54,58,59].

While it is generally accepted that Dectin-1 is able to induce Th17 responses [19] and instruct Treg to express IL-17 [60] *in vitro*, it is controversial whether Dectin-1 promotes these responses during a fungal infection. For example in mice Dectin-1 is not required for IL-17 production during a gastro-intestinal infection with *C. albicans* [61] but human deficiencies in Dectin-1 result in diminished Th17 responses and increased susceptibility to mucosal candidiasis [45]. This discrepancy reflects once more the existing differences among mice and men. Further, studies demonstrated that Dectin-1 recognition clearly depends on the fungal strains [62] and on the accessibility of the ligand on the fungal cell wall [63].

### 3.2. Dectin-2

An efficient antifungal immunity requires also Dectin-2, whose importance in protective anti-fungal immunity has been deeply investigated in mice [64,65]. While Dectin-1 recognizes the yeast fungal form, soluble Dectin-2 seems infact to preferentially bind the hyphal form of *C. albicans* [66], recognizing cell wall α-mannans [22]. Initially, Dectin-2 was identified as a Langerhans cell-specific receptor, but subsequently it was shown to be expressed on a variety of myeloid cells, including tissue macrophages, neutrophils and several DC subsets (e.g., pDCs) [20,67,68,69,70]. Dectin-2 shows a divergent expression pattern between humans and mice. In humans Dectin-2 [71,72] transcripts were detected in lung, spleen, lymph nodes, leukocytes, bone marrow and tonsils, but unlike in mice, Dectin-2 was not expressed in the human thymus.

While Dectin-1 forms a homodimer after engagement, Dectin-2 is associated to FcRγ [66]. Similar to Dectin-1, signaling from Dectin-2 passes through the Syk, PKCδ and CARD9–Bcl10–Malt1 pathway, leading to the induction of several cytokines and chemokines (including TNFα, IL-2, IL-10, IL-23, IL-1β, IL-6 and IL-12) [20,22,53,66,73] (Figure 3). Further in mice, Dectin-2 signaling has been shown to involve phospholipase Cγ2 (PLCγ2) and mitogen-activated protein kinases [74]. Here, *in vitro* experiments could unveil that, following fungal challenge, abrogating PLCγ2 results in the defective activation of NF-κB and MAPK and a significantly reduced production of reactive oxygen species. In addition, PLCγ2-deficient mice are defective in clearing *C. albicans* systemic infection *in vivo* [74]. Indeed, mice lacking Dectin-2 demonstrated greatly increased fungal susceptibility, with consequent increased fungal burden and death [22]. Abolishing Dectin-2 results in impaired cytokine response impacting the ability of the DCs to prime Th1/Th17 differentiation in response to *Candida* intraperitoneal infection [20,22]. Further, Dectin-2 promotes Th17-biased immunity in response to fungi through the differential activation of cRel containing NF-κB dimers by Malt1, and the preferential induction of IL-23 and IL-1β [75].

As observed for Dectin-1, triggering of the Dectin-2-Syk signaling induces the respiratory burst, fungal killing by ROS production [76] and NLRP3 inflammasome activation [77]. During *C. albicans* infection, Dectin-1 and Dectin-2 synergistically cooperate to cope with the invading fungus. Robinson *et al.* (2009) used a blocking mAb against Dectin-2 to treat murine wild-type and Dectin-1-deficient DCs. Stimulation of these DCs with heat-killed *C. albicans* or zymosan revealed an almost complete abolition of cytokine production when both of these CLRs were knocked out [20]. This result was reminiscent of those observed with Syk-deficient cells and therefore it has been proposed that all Syk-dependent responses to *Candida* may depend on Dectin-1 and Dectin-2. However, Th17 responses were only dependent on Dectin-2, and not Dectin-1, suggesting that Dectin-2 is the main receptor during *C. albicans* systemic infection [20].

### 3.3. Dectin-3

Several other microbial antigens contribute in sensing *C. albicans* [78,79,80,81,82,83]. Among those, Mincle, DCIR, BDCA2 and Dectin-3 (previously named CLECSF8 or Clec4d) have been shown to share structure and signaling features with Dectin-2, and therefore included in the Dectin-2 family [84,85,86]. Dectin-3 is expressed in peritoneal macrophages, in the spleen and bone marrow and only at lower level in lymph nodes and lungs [87]. Blocking or deficiency of Dectin-3 rendered mice more susceptible to *Candida* systemic infection [88] and Dectin-3 seems to have redundant functions with Dectin-2. Like Dectin-2 it is able to specifically recognize α-mannans that are exposed on the surface of *C. albicans* hyphae [88] and Dectin-3 stimulation by these hyphae effectively activates p65 nuclear translocation, Syk phosphorylation and Ikα degradation. Here, Syk inhibition completely blocks p65 nuclear translocation, assuming that Dectin-3 induces Syk-dependent activation of NF-κB [88]. This discovery could explain why murine cytokine production induced by *C. albicans* hyphae is only partially dependent on Dectin-2 [22]. In this regard, it is notable that a recent study demonstrated that Dectin-2 and Dectin-3 can form a heterodimeric PRR with increased sensitivities for recognizing surface mannose-rich structures [88].

### 3.4. Dectin-1 Antagonist: CLRs Antagonism

As shown above, some PRRs demonstrate some degree of redundancy in certain roles, which are not only restricted to CLRs. A well-known example of convergence and cooperation in activation of inflammatory gene expression is the integration between Dectin-1 and TLR2-mediated signaling. This synergism is dependent on Syk and is most pronounced during NF-κB activation [89]. It leads to a consequent production of Th17 priming cytokines like TNFα, IL-6, IL-23 [89] and IL-1β [59], but simultaneously to down-regulation of IL-12p70 [89]. This is coherent with the discovery that IL-12p70 is the target of Mincle suppression [55]. It has been recently shown that while Dectin-1 and Dectin-2 trigger the Syk-CARD9-MALT1-BCL10 module to activate NF-κB, Mincle uses the same signaling to induce a PI3K-PKB activation without NF-κB activation. The simultaneous activation of Dectin-1 and Mincle leads to DC induction of IL-6, IL-23, IL-1β but not IL-12p70. Mincle activates a secondary signaling through PI3K-PKB that directed Mdm2-mediated proteosomal degradation of IRF1. IRF1 degradation and subsequent selective abrogation of IL-12p35 transcription promote a shift from a Th1 antifungal response towards Th2 differentiation [55]. This shift probably contributes to the establishment of fungal infections, and thus, presents a strategy by which fungal pathogens exploit selective PRRs to evade and promote their survival.

## 4. The Mucosal and Epithelial Barrier and *C. albicans*

*C. albicans* is able to colonize the healthy oral, vaginal and gastro-intestinal mucosa and the skin. Here, immune defensive mechanisms allow colonization by *Candida* but prevent its dissemination and invasion, unless the epithelial barrier is breached or the immune system compromised. Indeed an equilibrium exists that balances the defense response against invading tissue. Moyes *et al.* [90] attempt to understand how epithelial cells, the cell type in closest contact with mucosal or skin flora, are able to distinguish between colonizing *C. albicans* that poses no apparent threat and the invading *Candida* that can result in mucosal or systemic infection. The authors proposed that a common core of recognition leads to the early activation of NF-κB and the MAPK c-Jun through p38 in a morphology independent manner. After this initial recognition hyphal formation and fungal burden can elicit a second signaling through MAPK, MKP1, and c-FOS which consequently induces a strong pro-inflammatory response. Thus, fungal germination and the recognition of hyphae by the epithelial cell represent a major danger signal upon tissue invasion. Nowadays a paucity of data exists on the role of receptors and signaling integration that enable the host immune system to discriminate between colonizing and invading *Candida*, revealing that Dectin-1 and Dectin-2 seem to be differently involved in different mucosal sites.

### 4.1. Skin

Mucocutaneous immunity to *C. albicans* requires Th17 differentiation that *in vitro* is activated upon hyphae recognition. On the skin stratum corneum, *C. albicans* exists as yeast form, while in the dermis and internal organs it is predominantly present as pathogenic pseudo-hyphae [91]. During epicutaneous infection, CD103^+^ dermal DCs prime Th1 differentiation through IL-12 secretion [92]. Langerhans cells (LCs) on the other hand are fundamental for Th17 differentiation via IL-1β, TGFβ and IL-6 production [93]. Of consequence, several subsets of DCs could be potentially engaged to properly respond to the various morphotypes. Using a skin infection mouse model, it has recently been shown that *C. albicans* yeasts but not pseudo-hyphae were able to induce Th17 adaptive responses. This activation relies on Dectin-1 triggering on LCs and subsequent IL-6 production. Nevertheless, pseudohyphae, that after spreading and invasion are the predominant form on the skin, failed to activate Dectin-1 competent CD11b^+^ dermal DCs [94]. This defect could be rescued by addition of exogenous Dectin-1 ligand, indicating that, during the epicutaneous challenge, dermal DCs have limited access to antigens due to *Candida* morphological switch [94]. The same authors found that Th17 provided protection against cutaneous but not systemic infection, while Th1 provided the opposite [94].

Innate immune cells with phagocytic activity such as neutrophils, macrophages and mast cells are the first defense line on the skin. Mast cells for example can degranulate in response to *C. albicans* and kill them [95] before they induce chemokines and cytokines release to recruit other immune effector cells [96]. Mast cells are equipped with a vast array of PRRs, including Dectin-1, whose role in mediating their response to *C. albicans* has been recently addressed [97]. It is now clear that mast cells are able to recognize both the hyphal and the yeast form, by inducing Syk phosphorylation and NF-κB activation through IKBα degradation. Phagocytosis and ROS production were associated with TNFα, IL-6, IL-10, CCL3 and CCL4. However, just the yeast form was able to induce IL-1β, through NLRP3 inflammasome activation [97]. NLRP3 plays an essential role in host defence during *in vivo* infection with *C. albicans* [59]. Moreover, the switch from yeast to hyphae is necessary for NLRP3 activation and IL-1β secretion in macrophages, and it was suggested that the NLRP3 inflammasome is activated by the membrane disruption that occurs during this transition, rather than by the hyphae themselves [98]. This specific morphotype activation reveals once more the fundamental role of the morphological transition for *C. albicans*’ adaptation to the host.

### 4.2. Mouth

Candidosis is the most common oral opportunistic infection [99,100]. Symptomatic oral infection with *C. albicans* is characterized by invasion of the oral epithelium by virulent hyphae. In response to them, myeloid DCs in the oral mucosal epithelium activate different receptors including TLR2, Dectin-1 and Dectin-2. Their individual or combined activation by *C. albicans* hyphae, triggers intracellular signaling pathways including the NF-κB, the MAPK and the IRF pathways [90,101]. Like on other mucosa, *C. albicans*-induced activation of the Dectin-1 receptor-Syk-CARD9 intracellular signaling [19] and co-stimulation of Dectin-1 and Dectin-2 pathways [75] promote DCs maturation and secretion of IL-23 and IL-β, and thus consequently boosts the differentiation of Th17 cells. This adaptive response, rather than the Th1 response, appears essential in limiting the oral candidiasis infection in both human [99] and mice [100]. In humans, deficiency in CARD9, the central signaling molecule downstream of CLRs, determines susceptibility to chronic mucocutaneous candidiasis due to a lack of Th17 response induction [102]. In mice, it has been observed that CARD9 is required for the adaptive *C. albicans* response but not necessarily for effective oral mucosal innate protection carried out by IL-17 producing innate cells [103]. This discrepancy in innate and adaptive immune activation by CLR-mediated signaling could indicate how the immune system evolved to specifically respond to the infection requirements.

### 4.3. Gut

Fungi and particularly *C. albicans* are important components of the gut microbiota. In the gut, fungi are recognized by Dectin-1. Its absence seems to increase susceptibility to murine experimental colitis, leading to an increase in *Candida* colonization, and in parallel in humans a severe form of ulcerative colitis is strongly linked to a polymorphism in the gene for Dectin-1 (CLEC7A) [104].

Along the gastrointestinal tract from the ileum to the colon Dectin-1 has been shown to be expressed not only on the surface of immune cells but also on intestinal epithelial cells (IEC) [105]. Its expression was found similar to that presented by lamina propria CD45^+^ CD13^+^ macrophages. IEC activation by β-glucan leads to a Dectin-1-dependent Syk phosphorylation and cytokine chemokine production, resulting in high levels of IL-8 and CCL2 [105]. This recent discovery proposes that IEC could act in concert with DCs and resident macrophages in mucosal recognition and immunity to *C. albicans*. Moreover, other PRR signaling might induce Syk phosphorylation *in vivo* [106,107], thus the involvement of Dectin-2, Dectin-3 and Mincle cannot be excluded in IEC driven fungal immunity.

In susceptible hosts systemic *C. albicans* infections are thought to start in the gastro-intestinal tract. As hyphal forms predominate at sites of primary epithelial infection [90], morphogenetic shift back to yeast cells seems to facilitate the access of *C. albicans* to the bloodstream and their consequent systemic spread [91]. To test this hypothesis of GI colonization immunocompetent mice were treated with antibiotics and led to be colonized by hyphae or yeasts of *C. albicans*. The results showed that the yeast form was favored in the GI tract while the filamentous form was rapidly cleared [108]. In contrast, for tissue invasion Vautier *et al* [108] discovered that the ability to transition between morphotypes is essential for GI survival in conditional mouse mutants. This is in agreement with the discovery of the yeast-like GUT morphotype, present after gut colonization [40]. During systemic infections Dectin-1-deficient mice were in fact susceptible to localized GI tract colonization, correlated to increased fungal burdens, dysregulated cytokine responses and increased levels of bile acids. As a consequence, Dectin-1 might also be involved in controlling the colonization of gut mucosa by *C. albicans* following a previous oral infection. As mice are not colonized in a normal fashion by this fungal pathogen, similarly to the GI colonization experiment mice are treated with antibiotics to displace the endogenous microbiota and then orally infected with *C. albicans* via the drinking water [109]. Surprisingly, after oral infection, no difference in colonization levels of the Dectin-1^−/−^ mice, was observed when compared to wild-type animals, neither in stool burdens nor in GI tissue fungal burdens. Moreover, Dectin-1 deficiency did not affect cytokine responses or tissue pathology during fungal GI colonization [61], revealing that Dectin-1 is not required for controlling mucosal colonization of the GI but it is fundamental for controlling systemic infection [61,110].

### 4.4. Vagina

Approximately 75% of all adult women are subject to vulvovaginal candidiasis at least once in their lives, and approximately half of these cases are re-infected with a rate of 5%–8% resulting in recurrent vulvovaginal candidiasis [111]. This indicates that some women are more prone to vaginal candidiasis. Dectin-1 Y238X polymorphism was initially correlated to individual predisposition causing Candida colonization and candidemia in 142 patients, who had a hematological malignancy [112]. The relationship between recurrent vulvovaginal candidiasis and Dectin-1 Y238X polymorphism was recently investigated [113] in 100 patients. Among them one individual had homozygous Dectin-1 polymorphism and 13 individuals had heterozygous Dectin-1 polymorphism. This frequency could be defined as a polymorphism, however, the authors were not able to correlate the presence of Dectin-1 Y238X polymorphism with recurrent vulvovaginal candidiasis etiology [113]. This preliminary result suggests a potential absence of Dectin-1 requirement in vaginal mucosa defense. However, the analysis was restricted to only a single polymorphism in Dectin-1 gene, thus limiting any definitive conclusion.

It has been shown that during *Candida* vaginal infection, and in general mucosal candidiasis, Dectin-1 crucially contributes to the balance of Th1/Th17/Treg responses. However, the contribution of Dectin-1 mediated response is dependent on the genetic background of the host. In particular, different mice respond differently to mucosal candidiasis, in a manner dependent on Dectin-1 isoforms expression [114]. Macrophages from BALB/c mice express similar levels of the full-lenght Dectin-1A and the stalkless dectin-1B, while C57BL/6 derived macrophages predominantly express the smaller isoform [115]. This determines a different outcome in resistance and protection to the invading Candida, where BALB/c mice showed more resistance to re-infection. Dectin-1 deficiency disproportionally increases the Th17 cell-mediated response in C57BL/6 mice and the Th1/Treg balance in BALB/c mice. This last finding indicates that Dectin-1 signaling pathway could be involved in both Th1 and Th17 priming.

## 5. Conclusion

The shift between commensalism and pathogenicity is a combination of failure of host defenses and increased expression of specific virulence traits that promote establishment and progression of infection. The main driver of differential pathogenicity of different *C. albicans* strains seems to rely on the rapidity of the morphological switch and the resulting ability to evade immune defenses of the host. Especially, the different recognition of yeast and hyphae cells by the immune system prevents the drawing of a conclusive recognition model. Even though significant advancements regarding the molecular mechanisms underlying the morphological transition and the respective immune sensing, have been achieved in the last decade, those achievements are far from being conclusive and complete. This is mainly caused by contradictory reports to the prevailing view that yeast cells, but not *C. albicans* hyphae, trigger cytokine stimulation by human PBMCs and macrophages [116,117,118]. Here, new studies revealed that in contrast hyphae, but not yeast cells, stimulate macrophages’ production of IL-1β via the Nlrp3 inflammasome [98,119], a protective Th17 response [119], and an innate response in oral and vaginal epithelial cells [90,101].

Additionally, the failure of murine studies in unequivocally showing the role of Dectin-1 and Th17 response during systemic and mucosal *Candida* infection reveals (1) the possible involvement of different CLRs, (e.g., Dectin-2, Dectin-3); (2) how the confounding results obtained by using different models could rely on the site-specific requirements of peculiar cells to properly cope with the diverse *Candida* morphotypes; (3) how immune outcomes can be influenced by the strains used as infection agent.

Several studies are suggesting that variability in fungal immune reactivity could depend on cell wall composition. This cell wall plasticity reflects the different ability of strains to face hostile environmental conditions, including temperature, pH and carbon source availability.

Thus, we believe that the main future discoveries will stem from the investigation of the evolvability and ability of *C. albicans* to adapt to rapidly changing environments and to colonize diverse host microniches. The availability of a *C. albicans* deletion collection and of a large number of clinical isolates make *Candida* a perfect model to study the boundary between commensalism and pathogenicity, as a continuum between optimal recognition and host immune escape. In this respect, comparative population immunology studies should be addressed at understanding whether the ability of the immune system to discriminate between commensals and colonizers is specific or subtends a common scheme that can be applied to other less investigated pathogenic *Candida* species.

## References

[1] Oh J., Freeman A.F., Park M., Sokolic R., Candotti F., Holland S.M., Segre J.A., Kong H.H., NISC Comparative Sequencing Program (2013). The altered landscape of the human skin microbiome in patients with primary immunodeficiencies. Genome Res..

[2] Iliev I.D., Underhill D.M. (2013). Striking a Balance: Fungal Commensalism *versus* Pathogenesis. Curr. Opin. Microbiol..

[3] Findley K., Oh J., Yang J., Conlan S., Deming C., Meyer J.A., Schoenfeld D., Nomicos E., Park M., Kong H.H., Segre J.A., NIH Intramural Sequencing Center Comparative Sequencing Program (2013). Topographic diversity of fungal and bacterial communities in human skin. Nature.

[4] Ghannoum M.A., Jurevic R.J., Mukherjee P.K., Cui F., Sikaroodi M., Naqvi A., Gillevet P.M. (2010). Characterization of the oral fungal microbiome (mycobiome) in healthy individuals. PLoS Pathog..

[5] Drell T., Lillsaar T., Tummeleht L., Simm J., Aaspõllu A., Väin E., Saarma I., Salumets A., Donders G.G.G., Metsis M. (2013). Characterization of the vaginal micro- and mycobiome in asymptomatic reproductive-age Estonian women. PLoS ONE.

[6] Merenstein D., Hu H., Wang C., Hamilton P., Blackmon M., Chen H., Calderone R., Li D. (2013). Colonization by Candida species of the oral and vaginal mucosa in HIV-infected and noninfected women. AIDS Res. Hum. Retrovir..

[7] Hoffmann C., Dollive S., Grunberg S., Chen J., Li H., Wu G.D., Lewis J.D., Bushman F.D. (2013). Archaea and fungi of the human gut microbiome: Correlations with diet and bacterial residents. PLoS ONE.

[8] Perlroth J., Choi B., Spellberg B. (2007). Nosocomial fungal infections: Epidemiology, diagnosis, and treatment. Med. Mycol..

[9] Rizzetto L., De Filippo C., Cavalieri D. (2014). Richness and diversity of mammalian fungal communities shape innate and adaptive immunity in health and disease. Eur. J. Immunol..

[10] Mukherjee P.K., Chandra J., Retuerto M., Sikaroodi M., Brown R.E., Jurevic R., Salata R.A., Lederman M.M., Gillevet P.M., Ghannoum M.A. (2014). Oral mycobiome analysis of HIV-infected patients: Identification of Pichia as an antagonist of opportunistic fungi. PLoS Pathog..

[11] Charlson E.S., Diamond J.M., Bittinger K., Fitzgerald A.S., Yadav A., Haas A.R., Bushman F.D., Collman R.G. (2012). Lung-enriched organisms and aberrant bacterial and fungal respiratory microbiota after lung transplant. Am. J. Respir. Crit. Care Med..

[12] Delhaes L., Monchy S., Fréalle E., Hubans C., Salleron J., Leroy S., Prevotat A., Wallet F., Wallaert B., Dei-Cas E. (2012). The airway microbiota in cystic fibrosis: A complex fungal and bacterial community--implications for therapeutic management. PLoS ONE.

[13] WHO Antimicrobial Resistance: Global Report on Surveillance 2014. http://www.who.int/drugresistance/documents/surveillancereport/en/.

[14] Mochon A.B., Jin Y., Ye J., Kayala M.A., Wingard J.R., Clancy C.J., Nguyen M.H., Felgner P., Baldi P., Liu H. (2010). Serological profiling of a *Candida albicans* protein microarray reveals permanent host-pathogen interplay and stage-specific responses during candidemia. PLoS Pathog..

[15] Sendid B., Jouault T., Vitse A., Fradin C., Colombel J.F., Poulain D. (2009). Anti-glycan antibodies establish an unexpected link between C. albicans and Crohn disease. Méd. Sci. MS.

[16] Saville S.P., Lazzell A.L., Monteagudo C., Lopez-Ribot J.L. (2003). Engineered control of cell morphology *in vivo* reveals distinct roles for yeast and filamentous forms of *Candida albicans* during infection. Eukaryot. Cell.

[17] Wächtler B., Wilson D., Haedicke K., Dalle F., Hube B. (2011). From attachment to damage: Defined genes of *Candida albicans* mediate adhesion, invasion and damage during interaction with oral epithelial cells. PLoS ONE.

[18] Vautier S., MacCallum D.M., Brown G.D. (2012). C-type lectin receptors and cytokines in fungal immunity. Cytokine.

[19] LeibundGut-Landmann S., Gross O., Robinson M.J., Osorio F., Slack E.C., Tsoni S.V., Schweighoffer E., Tybulewicz V., Brown G.D., Ruland J. (2007). Syk- and CARD9-dependent coupling of innate immunity to the induction of T helper cells that produce interleukin 17. Nat. Immunol..

[20] Robinson M.J., Osorio F., Rosas M., Freitas R.P., Schweighoffer E., Gross O., Verbeek J.S., Ruland J., Tybulewicz V., Brown G.D. (2009). Dectin-2 is a Syk-coupled pattern recognition receptor crucial for Th17 responses to fungal infection. J. Exp. Med..

[21] Gringhuis S.I., Den Dunnen J., Litjens M., Van der Vlist M., Wevers B., Bruijns S.C.M., Geijtenbeek T.B.H. (2009). Dectin-1 directs T helper cell differentiation by controlling noncanonical NF-kappaB activation through Raf-1 and Syk. Nat. Immunol..

[22] Saijo S., Ikeda S., Yamabe K., Kakuta S., Ishigame H., Akitsu A., Fujikado N., Kusaka T., Kubo S., Chung S. (2010). Dectin-2 recognition of alpha-mannans and induction of Th17 cell differentiation is essential for host defense against *Candida albicans*. Immunity.

[23] Puel A., Cypowyj S., Bustamante J., Wright J.F., Liu L., Lim H.K., Migaud M., Israel L., Chrabieh M., Audry M. (2011). Chronic mucocutaneous candidiasis in humans with inborn errors of interleukin-17 immunity. Science.

[24] Marakalala M.J., Vautier S., Potrykus J., Walker L.A., Shepardson K.M., Hopke A., Mora-Montes H.M., Kerrigan A., Netea M.G., Murray G.I. (2013). Differential adaptation of *Candida albicans in vivo* modulates immune recognition by dectin-1. PLoS Pathog..

[25] Rizzetto L., Giovannini G., Bromley M., Bowyer P., Romani L., Cavalieri D. (2013). Strain dependent variation of immune responses to A. fumigatus: Definition of pathogenic species. PLoS ONE.

[26] Noble S.M., French S., Kohn L.A., Chen V., Johnson A.D. (2010). Systematic screens of a *Candida albicans* homozygous deletion library decouple morphogenetic switching and pathogenicity. Nat. Genet..

[27] Sudbery P.E. (2011). Growth of *Candida albicans* hyphae. Nat. Rev. Microbiol..

[28] Gantner B.N., Simmons R.M., Underhill D.M. (2005). Dectin-1 mediates macrophage recognition of *Candida albicans* yeast but not filaments. EMBO J..

[29] Wheeler R.T., Fink G.R. (2006). A drug-sensitive genetic network masks fungi from the immune system. PLoS Pathog..

[30] Brown G.D. (2011). Innate antifungal immunity: The key role of phagocytes. Annu. Rev. Immunol..

[31] Bain J.M., Louw J., Lewis L.E., Okai B., Walls C.A., Ballou E.R., Walker L.A., Reid D., Munro C.A., Brown A.J.P. (2014). *Candida albicans* Hypha Formation and Mannan Masking of β-Glucan Inhibit Macrophage Phagosome Maturation. MBio.

[32] O’Meara T.R., Veri A.O., Ketela T., Jiang B., Roemer T., Cowen L.E. (2015). Global analysis of fungal morphology exposes mechanisms of host cell escape. Nat. Commun..

[33] Lewis L.E., Bain J.M., Lowes C., Gillespie C., Rudkin F.M., Gow N.A.R., Erwig L.-P. (2012). Stage specific assessment of *Candida albicans* phagocytosis by macrophages identifies cell wall composition and morphogenesis as key determinants. PLoS Pathog..

[34] Butler G., Rasmussen M.D., Lin M.F., Santos M.A.S., Sakthikumar S., Munro C.A., Rheinbay E., Grabherr M., Forche A., Reedy J.L. (2009). Evolution of pathogenicity and sexual reproduction in eight Candida genomes. Nature.

[35] Bezerra A.R., Simões J., Lee W., Rung J., Weil T., Gut I.G., Gut M., Bayés M., Rizzetto L., Cavalieri D. (2013). Reversion of a fungal genetic code alteration links proteome instability with genomic and phenotypic diversification. Proc. Natl. Acad. Sci. USA..

[36] Miranda I., Silva-Dias A., Rocha R., Teixeira-Santos R., Coelho C., Gonçalves T., Santos M.A.S., Pina-Vaz C., Solis N.V., Filler S.G. (2013). *Candida albicans* CUG mistranslation is a mechanism to create cell surface variation. MBio.

[37] Morschhäuser J. (2010). Regulation of white-opaque switching in *Candida albicans*. Med. Microbiol. Immunol..

[38] Soll D.R. (2009). Why does *Candida albicans* switch?. FEMS Yeast Res..

[39] Tao L., Du H., Guan G., Dai Y., Nobile C.J., Liang W., Cao C., Zhang Q., Zhong J., Huang G. (2014). Discovery of a “White-Gray-Opaque” Tristable Phenotypic Switching System in *Candida albicans*: Roles of Non-genetic Diversity in Host Adaptation. PLoS Biol..

[40] Pande K., Chen C., Noble S.M. (2013). Passage through the mammalian gut triggers a phenotypic switch that promotes *Candida albicans* commensalism. Nat. Genet..

[41] Soll D.R. (2014). The evolution of alternative biofilms in an opportunistic fungal pathogen: An explanation for how new signal transduction pathways may evolve. Infect. Genet. Evol. J. Mol. Epidemiol. Evol. Genet. Infect. Dis..

[42] Finkel J.S., Mitchell A.P. (2011). Genetic control of *Candida albicans* biofilm development. Nat. Rev. Microbiol..

[43] Hardison S.E., Brown G.D. (2012). C-type lectin receptors orchestrate antifungal immunity. Nat. Immunol..

[44] Hara H., Ishihara C., Takeuchi A., Imanishi T., Xue L., Morris S.W., Inui M., Takai T., Shibuya A., Saijo S. (2007). The adaptor protein CARD9 is essential for the activation of myeloid cells through ITAM-associated and Toll-like receptors. Nat. Immunol..

[45] Ferwerda B., Ferwerda G., Plantinga T.S., Willment J.A., Van Spriel A.B., Venselaar H., Elbers C.C., Johnson M.D., Cambi A., Huysamen C. (2009). Human Dectin-1 deficiency and mucocutaneous fungal infections. N. Engl. J. Med..

[46] Ariizumi K., Shen G.L., Shikano S., Xu S., Ritter R., Kumamoto T., Edelbaum D., Morita A., Bergstresser P.R., Takashima A. (2000). Identification of a novel, dendritic cell-associated molecule, Dectin-1, by subtractive cDNA cloning. J. Biol. Chem..

[47] Goodridge H.S., Reyes C.N., Becker C.A., Katsumoto T.R., Ma J., Wolf A.J., Bose N., Chan A.S.H., Magee A.S., Danielson M.E. (2011). Activation of the innate immune receptor Dectin-1 upon formation of a “phagocytic synapse.”. Nature.

[48] Hernanz-Falcón P., Joffre O., Williams D.L., Reis e Sousa C. (2009). Internalization of Dectin-1 terminates induction of inflammatory responses. Eur. J. Immunol..

[49] Rosas M., Liddiard K., Kimberg M., Faro-Trindade I., McDonald J.U., Williams D.L., Brown G.D., Taylor P.R. (2008). The induction of inflammation by Dectin-1 *in vivo* is dependent on myeloid cell programming and the progression of phagocytosis. J. Immunol..

[50] Henson P.M. (1971). Interaction of cells with immune complexes: Adherence, release of constituents, and tissue injury. J. Exp. Med..

[51] Rizzetto L., De Filippo C., Rivero D., Riccadonna S., Beltrame L., Cavalieri D. (2013). Systems biology of host-mycobiota interactions: Dissecting Dectin-1 and Dectin-2 signalling in immune cells with DC-ATLAS. Immunobiology.

[52] Drummond R.A., Brown G.D. (2013). Signalling C-type lectins in antimicrobial immunity. PLoS Pathog..

[53] Strasser D., Neumann K., Bergmann H., Marakalala M.J., Guler R., Rojowska A., Hopfner K.-P., Brombacher F., Urlaub H., Baier G. (2012). Syk kinase-coupled C-type lectin receptors engage protein kinase C-σ to elicit Card9 adaptor-mediated innate immunity. Immunity.

[54] Goodridge H.S., Shimada T., Wolf A.J., Hsu Y.-M.S., Becker C.A., Lin X., Underhill D.M. (2009). Differential Use of CARD9 by Dectin-1 in Macrophages and Dendritic Cells. J. Immunol..

[55] Wevers B.A., Kaptein T.M., Zijlstra-Willems E.M., Theelen B., Boekhout T., Geijtenbeek T.B.H., Gringhuis S.I. (2014). Fungal engagement of the C-type lectin mincle suppresses Dectin-1-induced antifungal immunity. Cell Host Microbe.

[56] Del Fresno C., Soulat D., Roth S., Blazek K., Udalova I., Sancho D., Ruland J., Ardavín C. (2013). Interferon-β production via Dectin-1-Syk-IRF5 signaling in dendritic cells is crucial for immunity to C. albicans. Immunity.

[57] Jia X.-M., Tang B., Zhu L.-L., Liu Y.-H., Zhao X.-Q., Gorjestani S., Hsu Y.-M.S., Yang L., Guan J.-H., Xu G.-T. (2014). CARD9 mediates Dectin-1—Induced ERK activation by linking Ras-GRF1 to H-Ras for antifungal immunity. J. Exp. Med..

[58] Gringhuis S.I., Kaptein T.M., Wevers B.A., Theelen B., Van der Vlist M., Boekhout T., Geijtenbeek T.B.H. (2012). Dectin-1 is an extracellular pathogen sensor for the induction and processing of IL-1β via a noncanonical caspase-8 inflammasome. Nat. Immunol..

[59] Hise A.G., Tomalka J., Ganesan S., Patel K., Hall B.A., Brown G.D., Fitzgerald K.A. (2009). An essential role for the NLRP3 inflammasome in host defense against the human fungal pathogen *Candida albicans*. Cell Host Microbe.

[60] Osorio F., LeibundGut-Landmann S., Lochner M., Lahl K., Sparwasser T., Eberl G., Reis e Sousa C. (2008). DC activated via Dectin-1 convert Treg into IL-17 producers. Eur. J. Immunol..

[61] Vautier S., Drummond R.A., Redelinghuys P., Murray G.I., MacCallum D.M., Brown G.D. (2012). Dectin-1 is not required for controlling *Candida albicans* colonization of the gastrointestinal tract. Infect. Immun..

[62] Netea M.G., Gow N.A.R., Joosten L.A.B., Verschueren I., Van der Meer J.W.M., Kullberg B.J. (2010). Variable recognition of *Candida albicans* strains by TLR4 and lectin recognition receptors. Med. Mycol..

[63] Rizzetto L., Buschow S.I., Beltrame L., Figdor C.G., Schierer S., Schuler G., Cavalieri D. (2012). The modular nature of dendritic cell responses to commensal and pathogenic fungi. PLoS ONE.

[64] Ifrim D.C., Bain J.M., Reid D.M., Oosting M., Verschueren I., Gow N.A.R., Van Krieken J.H., Brown G.D., Kullberg B.-J., Joosten L.A.B. (2014). Role of Dectin-2 for Host Defense against Systemic Infection with Candida glabrata. Infect. Immun..

[65] Kerscher B., Willment J.A., Brown G.D. (2013). The Dectin-2 family of C-type lectin-like receptors: An update. Int. Immunol..

[66] Sato K., Yang X., Yudate T., Chung J.-S., Wu J., Luby-Phelps K., Kimberly R.P., Underhill D., Cruz P.D., Ariizumi K. (2006). Dectin-2 is a pattern recognition receptor for fungi that couples with the Fc receptor gamma chain to induce innate immune responses. J. Biol. Chem..

[67] Ariizumi K., Shen G.L., Shikano S., Ritter R., Zukas P., Edelbaum D., Morita A., Takashima A. (2000). Cloning of a second dendritic cell-associated C-type lectin (Dectin-2) and its alternatively spliced isoforms. J. Biol. Chem..

[68] Taylor P.R., Reid D.M., Heinsbroek S.E.M., Brown G.D., Gordon S., Wong S.Y.C. (2005). Dectin-2 is predominantly myeloid restricted and exhibits unique activation-dependent expression on maturing inflammatory monocytes elicited *in vivo*. Eur. J. Immunol..

[69] Seeds R.E., Gordon S., Miller J.L. (2009). Characterisation of myeloid receptor expression and interferon alpha/beta production in murine plasmacytoid dendritic cells by flow cytomtery. J. Immunol. Methods.

[70] McDonald J.U., Rosas M., Brown G.D., Jones S.A., Taylor P.R. (2012). Differential dependencies of monocytes and neutrophils on Dectin-1, Dectin-2 and complement for the recognition of fungal particles in inflammation. PLoS ONE.

[71] Gavino A.C.P., Chung J.-S., Sato K., Ariizumi K., Cruz P.D. (2005). Identification and expression profiling of a human C-type lectin, structurally homologous to mouse Dectin-2. Exp. Dermatol..

[72] Kanazawa N., Tashiro K., Inaba K., Lutz M.B., Miyachi Y. (2004). Molecular cloning of human Dectin-2. J. Invest. Dermatol..

[73] Bi L., Gojestani S., Wu W., Hsu Y.-M.S., Zhu J., Ariizumi K., Lin X. (2010). CARD9 mediates Dectin-2-induced IkappaBalpha kinase ubiquitination leading to activation of NF-kappaB in response to stimulation by the hyphal form of *Candida albicans*. J. Biol. Chem..

[74] Gorjestani S., Yu M., Tang B., Zhang D., Wang D., Lin X. (2011). Phospholipase Cγ2 (PLCγ2) is key component in Dectin-2 signaling pathway, mediating anti-fungal innate immune responses. J. Biol. Chem..

[75] Gringhuis S.I., Wevers B.A., Kaptein T.M., Van Capel T.M.M., Theelen B., Boekhout T., De Jong E.C., Geijtenbeek T.B.H. (2011). Selective C-Rel activation via Malt1 controls anti-fungal T(H)-17 immunity by Dectin-1 and Dectin-2. PLoS Pathog..

[76] Sun H., Xu X., Tian X., Shao H., Wu X., Wang Q., Su X., Shi Y. (2014). Activation of NF-κB and respiratory burst following Aspergillus fumigatus stimulation of macrophages. Immunobiology.

[77] Ritter M., Gross O., Kays S., Ruland J., Nimmerjahn F., Saijo S., Tschopp J., Layland L.E., Prazeres da Costa C. (2010). Schistosoma mansoni triggers Dectin-2, which activates the Nlrp3 inflammasome and alters adaptive immune responses. Proc. Natl. Acad. Sci. USA..

[78] Bugarcic A., Hitchens K., Beckhouse A.G., Wells C.A., Ashman R.B., Blanchard H. (2008). Human and mouse macrophage-inducible C-type lectin (Mincle) bind *Candida albicans*. Glycobiology.

[79] Cambi A., Gijzen K., De Vries L.J.M., Torensma R., Joosten B., Adema G.J., Netea M.G., Kullberg B.-J., Romani L., Figdor C.G. (2003). The C-type lectin DC-SIGN (CD209) is an antigen-uptake receptor for *Candida albicans* on dendritic cells. Eur. J. Immunol..

[80] Taylor P.R., Brown G.D., Herre J., Williams D.L., Willment J.A., Gordon S. (2004). The role of SIGNR1 and the beta-glucan receptor (Dectin-1) in the nonopsonic recognition of yeast by specific macrophages. J. Immunol..

[81] Van de Veerdonk F.L., Marijnissen R.J., Kullberg B.J., Koenen H.J.P.M., Cheng S.-C., Joosten I., Van den Berg W.B., Williams D.L., Van der Meer J.W.M., Joosten L.A.B. (2009). The macrophage mannose receptor induces IL-17 in response to *Candida albicans*. Cell Host Microbe.

[82] Wells C.A., Salvage-Jones J.A., Li X., Hitchens K., Butcher S., Murray R.Z., Beckhouse A.G., Lo Y.-L.-S., Manzanero S., Cobbold C. (2008). The macrophage-inducible C-type lectin, mincle, is an essential component of the innate immune response to *Candida albicans*. J. Immunol..

[83] Osorio F., Reis e Sousa C. (2011). Myeloid C-type lectin receptors in pathogen recognition and host defense. Immunity.

[84] Arce I., Martínez-Muñoz L., Roda-Navarro P., Fernández-Ruiz E. (2004). The human C-type lectin CLECSF8 is a novel monocyte/macrophage endocytic receptor. Eur. J. Immunol..

[85] Graham L.M., Brown G.D. (2009). The Dectin-2 family of C-type lectins in immunity and homeostasis. Cytokine.

[86] Kingeter L.M., Lin X. (2012). C-type lectin receptor-induced NF-κB activation in innate immune and inflammatory responses. Cell. Mol. Immunol..

[87] Balch S.G., McKnight A.J., Seldin M.F., Gordon S. (1998). Cloning of a novel C-type lectin expressed by murine macrophages. J. Biol. Chem..

[88] Zhu L.-L., Zhao X.-Q., Jiang C., You Y., Chen X.-P., Jiang Y.-Y., Jia X.-M., Lin X. (2013). C-type lectin receptors Dectin-3 and Dectin-2 form a heterodimeric pattern-recognition receptor for host defense against fungal infection. Immunity.

[89] Dennehy K.M., Willment J.A., Williams D.L., Brown G.D. (2009). Reciprocal regulation of IL-23 and IL-12 following co-activation of Dectin-1 and TLR signaling pathways. Eur. J. Immunol..

[90] Moyes D.L., Runglall M., Murciano C., Shen C., Nayar D., Thavaraj S., Kohli A., Islam A., Mora-Montes H., Challacombe S.J. (2010). A biphasic innate immune MAPK response discriminates between the yeast and hyphal forms of *Candida albicans* in epithelial cells. Cell Host Microbe.

[91] Gow N.A.R., Van de Veerdonk F.L., Brown A.J.P., Netea M.G. (2011). *Candida albicans* morphogenesis and host defence: Discriminating invasion from colonization. Nat. Rev. Microbiol..

[92] Igyártó B.Z., Haley K., Ortner D., Bobr A., Gerami-Nejad M., Edelson B.T., Zurawski S.M., Malissen B., Zurawski G., Berman J. (2011). Skin-resident murine dendritic cell subsets promote distinct and opposing antigen-specific T helper cell responses. Immunity.

[93] Hu W., Troutman T.D., Edukulla R., Pasare C. (2011). Priming microenvironments dictate cytokine requirements for T helper 17 cell lineage commitment. Immunity.

[94] Kashem S.W., Igyártó B.Z., Gerami-Nejad M., Kumamoto Y., Mohammed J., Jarrett E., Drummond R.A., Zurawski S.M., Zurawski G., Berman J. (2015). *Candida albicans* morphology and dendritic cell subsets determine T helper cell differentiation. Immunity.

[95] Trevisan E., Vita F., Medic N., Soranzo M.R., Zabucchi G., Borelli V. (2014). Mast cells kill *Candida albicans* in the extracellular environment but spare ingested fungi from death. Inflammation.

[96] St John A.L., Abraham S.N. (2013). Innate immunity and its regulation by mast cells. J. Immunol..

[97] Nieto-Patlán A., Campillo-Navarro M., Rodríguez-Cortés O., Muñoz-Cruz S., Wong-Baeza I., Estrada-Parra S., Estrada-García I., Serafín-López J., Chacón-Salinas R. (2015). Recognition of *Candida albicans* by Dectin-1 induces mast cell activation. Immunobiology.

[98] Joly S., Ma N., Sadler J.J., Soll D.R., Cassel S.L., Sutterwala F.S. (2009). Cutting Edge: *Candida albicans* Hyphae Formation Triggers Activation of the Nlrp3 Inflammasome. J. Immunol..

[99] Conti H.R., Shen F., Nayyar N., Stocum E., Sun J.N., Lindemann M.J., Ho A.W., Hai J.H., Yu J.J., Jung J.W. (2009). Th17 cells and IL-17 receptor signaling are essential for mucosal host defense against oral candidiasis. J. Exp. Med..

[100] Hernández-Santos N., Huppler A.R., Peterson A.C., Khader S.A., McKenna K.C., Gaffen S.L. (2013). Th17 cells confer long-term adaptive immunity to oral mucosal *Candida albicans* infections. Mucosal Immunol..

[101] Moyes D.L., Naglik J.R. (2011). Mucosal immunity and *Candida albicans* infection. Clin. Dev. Immunol..

[102] Glocker E.-O., Hennigs A., Nabavi M., Schäffer A.A., Woellner C., Salzer U., Pfeifer D., Veelken H., Warnatz K., Tahami F. (2009). A homozygous CARD9 mutation in a family with susceptibility to fungal infections. N. Engl. J. Med..

[103] Bishu S., Hernández-Santos N., Simpson-Abelson M.R., Huppler A.R., Conti H.R., Ghilardi N., Mamo A.J., Gaffen S.L. (2014). The Adaptor CARD9 Is Required for Adaptive but Not Innate Immunity to Oral Mucosal *Candida albicans* Infections. Infect. Immun..

[104] Iliev I.D., Funari V.A., Taylor K.D., Nguyen Q., Reyes C.N., Strom S.P., Brown J., Becker C.A., Fleshner P.R., Dubinsky M. (2012). Interactions between commensal fungi and the C-type lectin receptor Dectin-1 influence colitis. Science.

[105] Cohen-Kedar S., Baram L., Elad H., Brazowski E., Guzner-Gur H., Dotan I. (2014). Human intestinal epithelial cells respond to β-glucans via Dectin-1 and Syk. Eur. J. Immunol..

[106] Lowell C.A. (2011). Src-family and Syk kinases in activating and inhibitory pathways in innate immune cells: Signaling cross talk. Cold Spring Harb. Perspect. Biol..

[107] Mócsai A., Ruland J., Tybulewicz V.L.J. (2010). The SYK tyrosine kinase: A crucial player in diverse biological functions. Nat. Rev. Immunol..

[108] Vautier S., Drummond R.A., Chen K., Murray G.I., Kadosh D., Brown A.J.P., Gow N.A.R., MacCallum D.M., Kolls J.K., Brown G.D. (2015). *Candida albicans* colonization and dissemination from the murine gastrointestinal tract: The influence of morphology and Th17 immunity. Cell. Microbiol..

[109] Koh A.Y., Köhler J.R., Coggshall K.T., Van Rooijen N., Pier G.B. (2008). Mucosal damage and neutropenia are required for *Candida albicans* dissemination. PLoS Pathog..

[110] Taylor P.R., Tsoni S.V., Willment J.A., Dennehy K.M., Rosas M., Findon H., Haynes K., Steele C., Botto M., Gordon S. (2007). Dectin-1 is required for beta-glucan recognition and control of fungal infection. Nat. Immunol..

[111] Ferrer J. (2000). Vaginal candidosis: Epidemiological and etiological factors. Int. J. Gynaecol. Obstet. Off. Organ Int. Fed. Gynaecol. Obstet..

[112] Plantinga T.S., Van der Velden W.J.F.M., Ferwerda B., Van Spriel A.B., Adema G., Feuth T., Donnelly J.P., Brown G.D., Kullberg B.-J., Blijlevens N.M.A. (2009). Early stop polymorphism in human DECTIN-1 is associated with increased candida colonization in hematopoietic stem cell transplant recipients. Clin. Infect. Dis. Off. Publ. Infect. Dis. Soc. Am..

[113] Usluogullari B., Gumus I., Gunduz E., Kaygusuz I., Simavli S., Acar M., Oznur M., Gunduz M., Kafali H. (2014). The role of Human Dectin-1 Y238X Gene Polymorphism in recurrent vulvovaginal candidiasis infections. Mol. Biol. Rep..

[114] Carvalho A., Giovannini G., De Luca A., D’Angelo C., Casagrande A., Iannitti R.G., Ricci G., Cunha C., Romani L. (2012). Dectin-1 isoforms contribute to distinct Th1/Th17 cell activation in mucosal candidiasis. Cell. Mol. Immunol..

[115] Heinsbroek S.E.M., Taylor P.R., Rosas M., Willment J.A., Williams D.L., Gordon S., Brown G.D. (2006). Expression of functionally different Dectin-1 isoforms by murine macrophages. J. Immunol..

[116] Rogers N.C., Slack E.C., Edwards A.D., Nolte M.A., Schulz O., Schweighoffer E., Williams D.L., Gordon S., Tybulewicz V.L., Brown G.D. (2005). Syk-Dependent Cytokine Induction by Dectin-1 Reveals a Novel Pattern Recognition Pathway for C Type Lectins. Immunity.

[117] Dillon S., Agrawal S., Banerjee K., Letterio J., Denning T.L., Oswald-Richter K., Kasprowicz D.J., Kellar K., Pare J., Van Dyke T. (2006). Yeast zymosan, a stimulus for TLR2 and Dectin-1, induces regulatory antigen-presenting cells and immunological tolerance. J. Clin. Invest..

[118] Van der Graaf C.A.A., Netea M.G., Verschueren I., Van der Meer J.W.M., Kullberg B.J. (2005). Differential cytokine production and Toll-like receptor signaling pathways by *Candida albicans* blastoconidia and hyphae. Infect. Immun..

[119] Cheng S.-C., Van de Veerdonk F.L., Lenardon M., Stoffels M., Plantinga T., Smeekens S., Rizzetto L., Mukaremera L., Preechasuth K., Cavalieri D. (2011). The Dectin-1/inflammasome pathway is responsible for the induction of protective T-helper 17 responses that discriminate between yeasts and hyphae of *Candida albicans*. J. Leukoc. Biol..

